# The interwoven fibril-like structure of amyloid-beta plaques in mouse brain tissue visualized using super-resolution STED microscopy

**DOI:** 10.1186/s13578-023-01086-4

**Published:** 2023-08-04

**Authors:** Björn Johansson, Sho Oasa, Aida Muntsant Soria, Ann Tiiman, Linda Söderberg, Ebba Amandius, Christer Möller, Lars Lannfelt, Lars Terenius, Lydia Giménez-Llort, Vladana Vukojević

**Affiliations:** 1 Department of Clinical Neuroscience, Karolinska Institutet, SE-17176 Stockholm, Sweden; 2 Theme Aging, Karolinska University Hospital, Karolinska Institutet, SE-17176 Stockholm, Sweden; 3 Institut de Neurociències, Universitat Autònoma de Barcelona, 08193 Barcelona, Spain; 4 Department of Psychiatry and Forensic Medicine, School of Medicine, Universitat Autònoma de Barcelona, 08193 Barcelona, Spain; 5 BioArctic AB, Stockholm, Sweden

**Keywords:** Alzheimer’s disease, Amyloid beta peptide, Amyloid fibrils, Immunohistochemistry, Super-resolution STED microscopy, Next generation drug design

## Abstract

**Background:**

Standard neuropathologic analysis of Alzheimer’s brain relies on traditional fluorescence microscopy, which suffers from limited spatial resolution due to light diffraction. As a result, it fails to reveal intricate details of amyloid plaques. While electron microscopy (EM) offers higher resolution, its extensive sample preparation, involving fixation, dehydration, embedding, and sectioning, can introduce artifacts and distortions in the complex brain tissue. Moreover, EM lacks molecular specificity and has limited field of view and imaging depth.

**Results:**

In our study, we employed super-resolution Stimulated Emission Depletion (STED) microscopy in conjunction with the anti-human APP recombinant antibody 1C3 fluorescently labelled with DyLight^TM^633 (1C3-DyLight633). This combination allowed us to visualize amyloidogenic aggregates in vitro and in brain sections from a 17-month-old 3×Tg-AD mouse with sub-diffraction limited spatial resolution. Remarkably, we achieved a spatial resolution of 29 nm in vitro and 62 nm in brain tissue sections, surpassing the capabilities of conventional confocal microscopy by 5–10 times. Consequently, we could discern individual fibrils within plaques, an achievement previously only possible with EM.

**Conclusions:**

The utilization of STED microscopy represents a groundbreaking advancement in the field, enabling researchers to delve into the characterization of local mechanisms that underlie Amyloid (Aβ) deposition into plaques and their subsequent clearance. This unprecedented level of detail is especially crucial for comprehending the etiology of Alzheimer’s disease and developing the next generation of anti-amyloid treatments. By facilitating the evaluation of drug candidates and non-pharmacological interventions aiming to reduce amyloid burden, STED microscopy emerges as an indispensable tool for driving scientific progress in Alzheimer’s research.

## Introduction

The major component of plaques found in the brains of patients with Alzheimer´s disease (AD) are 40–42 amino acids long amyloid beta (Aβ) peptides derived from the amyloid precursor protein (APP) by enzymatic cleavage, first with β-secretase (BACE1) and then by γ-secretase [1, 2]. Immunohistochemistry has significantly contributed to mapping the distribution of Aβ peptides and Aβ amyloidogenic fibrils in the brain, both at the inter- and intracellular level. For example, Aβ_42_ was found in the nuclear envelope and endoplasmic reticulum, whereas Aβ_40_ was found to be restricted to the trans-Golgi network [3]. It was early on observed that the intracellular pathology becomes less evident as the extracellular Aβ deposition progresses, which has led D’Andrea to propose that intracellular Aβ can contribute to the generation of amyloid plaques in the human brain [4]. More precisely, using antibodies against Aβ_40_ and Aβ_42_, it was found that Aβ_42_ accumulated in granular bodies inside pyramidal neurons of AD brains [5]. Based on observations of an inverse relationship between plaque density and pyramidal neuron density, chromatin abnormalities in pyramidal neurons rich in Aβ_42_, larger intracellular Aβ_42_ granules in areas with higher plaque density and nuclear remnants in the dense core of plaques, it was suggested that Aβ_42_ amyloid fibrils first accumulate inside the neurons which eventually die, releasing their amyloid content from which the extracellular plaques are being formed in due course [4]. This notion of an intracellular pathology preceding extracellular plaque deposition was also supported by an independent immunocytochemical study by Gouras et al. [6] showing an age-dependent accumulation of intraneuronal Aβ_42_ in non-AD subjects, especially in AD-vulnerable brain regions.

Electron microscopy (EM) has often been used to examine the structure and morphology of Aβ aggregates and aggregates composed of other amyloidogenic peptides. Aggregates originating from ex vivo materials and from synthetic peptides/proteins in vitro, have appeared to be similarly elongated (thread-like), unbranching, and of a comparable diameter (6–10 nm) [7], often consisting of filaments wound around one another [8]. However, using light microscopy, which has limited spatial resolution of 220 nm at best, the pathological Aβ-deposits observed in the brain tissue could only be described in collective terms, e.g. as amorphous, dense core or diffuse plaques [9–11], without much relation to the individual Aβ fibres seen with EM that were shown to develop protease resistance [12].

To specifically visualize the fibril network structure of Aβ amyloid plaques in brain tissue with improved spatial resolution, we have resorted to Stimulated Emission Depletion (STED) microscopy. STED microscopy was first experimentally realized at the turn of the millennium by the group of S.W. Hell [13], as the first optical super-resolution technology allowing imaging with a spatial resolution that goes beyond the barrier imposed by the diffraction of light. STED images are constructed by scanning across the sample two concentric lasers, a focused excitation laser beam that is of the highest intensity at the centre and the so-called STED laser beam that is of a longer wavelength and has lowest intensity at its centre and highest at its circumference. The STED laser forces the excited molecules that are localized outside its centre to lose energy through stimulated emission and return to the ground state before being able to emit fluorescence. In this way, spatial resolution down to tens of nanometers was achieved in live cells when small molecular probes such as silicon-rhodamine (SiR) or germano-rhodamine (GeR), aptamers (≈ 15 kDa; ≈ 4 nm) or nanobodies (≈ 13 kDa; ≈ 2–4 nm) are being used [14]. For immunostaining, the spatial resolution is somewhat lower due to antibody size (≈ 150 kDa; 10–15 nm) and even lower when a combination of a primary and a fluorescently labelled secondary antibody is being used – here the labelling complex becomes ≈ 30 nm and the probes often cannot bind to every target molecule due to spatial constraints, giving rise to “spotty” images and a spatial resolution that is ≈ 40 nm at best [14].

Thus far, STED microscopy has only been used in a handful of AD-related studies, e.g., to visualize amyloid fibrils in vitro [15]; characterize in the cerebrospinal fluid (CSF) of individuals with AD the number and size of Aβ and tau aggregates [16]; determine the localization of γ-secretase in the neuronal synapse in mouse hippocampal neurons in culture [17–19]; examine nanoscale features of spine morphology in the APP/PS1 mouse model of AD amyloidosis [20]; and visualize normal, unaggregated tau protein in the mouse brain [21]. Querol-Vilaseca et al. [22] used super-resolution in three dimensions by Array Tomography (AT) and STED microscopy, to characterize non-fibrillar Aβ structures in amyloid plaques in post-mortem human brain tissue of AD, revealing that an amyloid plaque is formed by a dense core of higher order Aβ species (22 nm^3^) and a peripheral halo of smaller Aβ structures (3 nm^3^); whereas Hernández et al. combined STED with selective plane illumination microscopy (STED-SPIM) to image AD-related brain pathology with improved optical slicing [23]. In relation to other amyloid diseases, STED microscopy of cellular uptake of α-synuclein oligomers, putative causative agents in Parkinson’s disease, was recently described [24] and STED imaging of a Thioflavin T labelled amyloid of an α-synuclein mutant was developed [25]; STED was used to characterize huntingtin aggregates and sequestration in inclusion bodies [26], and to visualize apoferritin amyloid fibrils formation [27].

Recent success in the development of immunotherapies against Aβ for the treatment of AD [28] have renewed the interest in characterising the affinity profile and binding kinetics of monoclonal antibody drug candidates [29, 30], and have also highlighted the need for characterizing local mechanisms through which plaque formation/clearance is achieved. In this study, we show that STED microscopy allows us to visualize individual fibrils in plaques in brain tissue sections at a spatial resolution that is 5–10 times better than using conventional confocal microscopy.

## Materials and methods

### Animals and brain sections

Experiments were performed in accordance with the relevant guidelines from the Swedish National Board for Laboratory Animals, the Spanish legislation and the European Community Council Directive (2010/63/UE) on this subject under the protocol CEEAH 3588/DMAH 9452. The present study includes the analysis of brain sections from one 17-month-old 3×Tg-AD mouse bearing three human mutant genes: presenilin-1 (PS1) with the M146V mutation, the human APP gene with the Swedish mutation, and tau with the P301L mutation [31]. Mice were kept under standard laboratory conditions at Universitat Autònoma de Barcelona, with food and water ad libitum, T = (22 ± 2) °C, under a 12:12 h light: dark cycle and relative humidity of 40–60%. Euthanasia was performed using CO_2_. The brains were quickly dissected and immediately frozen on dry ice, followed by storage at −80 °C. Sagittal sections of 16 μm thickness were cut using a cryostat (Leica Jung CM 3000, Leica Microsystems, Wetzlar, Germany) at approximately −20 °C. Sections were collected on Superfrost Plus glass slides (Gerhard Menzel GmbH, Braunschweig, Germany) and stored at −20 °C. Sections cut at 2.88–2.90 mm from brain’s midline were selected and processed for immunohistochemistry as described below.

### Antibodies

Monoclonal anti-human APP recombinant antibody 1C3 that detects the linear N-terminal fragment comprising residues 2–8 and is not conformation specific [32] fluorescently labelled with DyLight^TM^ 633.

### Immunohistochemistry

For immunohistochemistry, brain tissue sections were blocked for 30 min with 10% horse serum, 5 mg/ml bovine serum albumin and 0.2% Triton X-100 in PBS. For amyloid plaque visualization, AD brain tissue sections were immunostained for 1 h using 500 nM 1C3-DyLight633 (BioArctic, Stockholm, Sweden). In immunohistochemistry control experiments, 1C3-DyLight633 binding was blocked by simultaneous co-incubation with unlabelled Aβ_40_ in large excess, 100 µg/ml (23 µM). For nuclear staining, the ProLong™ Gold Antifade Mountant with DAPI (ThermoFisher, P36935) was used following manufacturer’s instructions.

### Aβ_40_ aggregation in vitro

50 µg of the human recombinant Aβ_40_ peptide (Alexo-Tech AB, Umeå, Sweden) was dissolved in 50 µl of 10 mM NaOH and incubated at room temperature for 1 min. The peptide/NaOH solution was diluted to 10 µM peptide concentration with 20 mM HEPES buffer (pH 7.4) and incubated at room temperature for 1 h while stirring at 1100 rpm. After turning off the stirrer, the sample was allowed to rest and a 5 µl aliquot of Aβ_40_ aggregates that accumulated at the bottom of the reaction vessel was pipetted out and transferred to the grid for EM imaging. For STED microscopy, a 100 µl aliquot of Aβ_40_ aggregates that accumulated at the bottom of the reaction vessel was pipetted out, mixed with 1C3-DyLight633 to a 1C3-DyLight633 concentration of 500 nM, transferred to a #1.5 coverglass (VWR, 631 − 0136) and imaged. The Aβ_40_ aggregates were always freshly prepared before imaging.

### Transmission Electron Microscopy (TEM) imaging

A formvar coated TEM grid stabilized with evaporated carbon film on 200 mesh copper (Formvar/Carbon Film coated, 200 Mesh, Cu) was first hydrophilized by treatment in an EMS 100× glow discharge unit for 45 s at the current of 25 mA. Thereafter, a 5 µl aliquot of freshly prepared Aβ_40_ aggregates was transferred to the grid and incubated for 1 min at room temperature. The droplet was removed with a pipette and the specimen was negatively stained following the procedure described by Keller et al. [33]. Briefly, a 5 µl droplet of freshly prepared 1% uranyl acetate (UAc) was applied to the grid and incubated for a few seconds. The UAc droplet was removed and a fresh 5 µl UAc droplet was applied. The application-removal cycle was repeated seven times. Following the removal of the last droplet, the sample was air-dried for several minutes and subjected to TEM imaging using a Talos L120C transmission electron microscope (Thermo Fisher Scientific) operating at 120 kV. The images were acquired using a Ceta-D camera.

### Confocal laser scanning Microscopy (CLSM) imaging of whole brain tissue sections

CLSM imaging was performed using an LSM880 (Carl Zeiss) microscope system equipped with a 633 nm He-Ne laser, objective lens (Plan-Apochromat 10×/N.A. 0.45 M27), and a 32-channel gallium arsenide phosphide (GaAsP) spectral detector. DyLight^TM^633 fluorescence was excited using the 633 nm HeNe laser. The pinhole size was 90 μm. Fluorescence was spectrally split by gratings and detected in the 638–755 nm range by the GaAsP detector. The tile scan function was used to acquire an image series of neighboring fields of view and construct images of the whole mouse brain tissue section (size: 9 mm × 7 mm).

### Stimulated Emission Depletion (STED) microscopy imaging

Super-resolution STED and related CLSM images were acquired at the same position using the STEDYCON compact line nanoscope (Abberior Instruments GmbH, Göttingen, Germany) mounted on an Zeiss Axio Imager Z2 (Carl Zeiss) microscope. The STEDYCON nanoscope unit is equipped with a 405 nm continuous wavelength laser, a 640 nm pulsed excitation laser and a 775 nm pulsed STED laser; oil immersion objective (Plan-Apochromat 100×/N.A. 1.46 Oil DIC M27) and avalanche diode detectors (APDs). DAPI and DyLight633 fluorescence were excited using the 405 nm and the 640 nm lasers, respectively. The pinhole size was 64 μm. The fluorescence was split and detected by distinct APDs (DAPI: 420–475 nm, DyLight633: 650–700 nm). The STED laser power was set to 98% (maximum power) to acquire images with the highest spatial resolution possible.

## Results

### Super-resolution STED imaging of in vitro formed Aβ_40_ aggregates

**Fig. 1:**
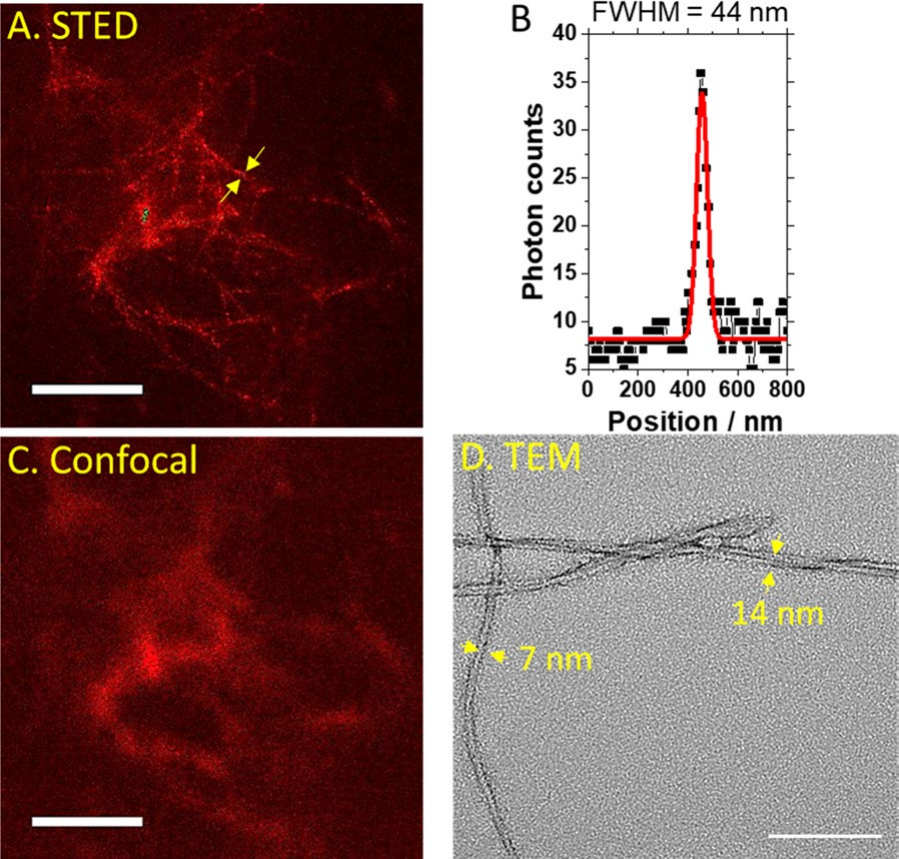
Super-resolution STED microscopy and TEM images of in vitro formed Aβ_40_ aggregates. **A** Super-resolution STED microscopy image of in vitro formed Aβ_40_ aggregates rendered visible using 1C3-DyLight633. Scale bar 2 μm. **B** Fluorescence intensity profile along the fibril highlighted by yellow arrows showing a fibril diameter of 44 nm. **C** The diffraction-limited CLSM image of Aβ_40_ aggregates shown in A. Scale bar 2 μm. **D** Negatively stained TEM image of in vitro formed Aβ_40_ aggregates showing single-threaded filaments (7 ± 2) nm and twisted two-filament fibers (14 ± 1) nm. Scale bar 100 nm

A STED image of Aβ_40_ aggregates formed in vitro and immune-stained using the antibody 1C3-DyLight633 (Fig. 1A), showed spatially well-resolved fibrils (Fig. 1A and B) that could not be easily discerned in the diffraction-limited image acquired by conventional confocal microscopy (Fig. 1C). TEM imaging (Fig. 1D), confirmed the STED microscopy findings, convincingly showing that most of the precipitated aggregates are either single-thread filaments or twisted two-thread filaments (Fig. 1D). The smallest filament thickness, 29 nm, and average fibril thickness, (44 ± 13) nm, were discernable by STED microscopy, as evident from the full width at half-maximum (FWHM) of the fluorescence intensity distribution across the fibril (Fig. 1B). Given the antibody size (10–15 nm) and the thickness of the filament/fibril (7–14 nm), this value is in good agreement with the true size of the fibril–antibody complex.

### Super-resolution STED imaging of Aβ aggregates in brain tissue sections

**Fig. 2:**
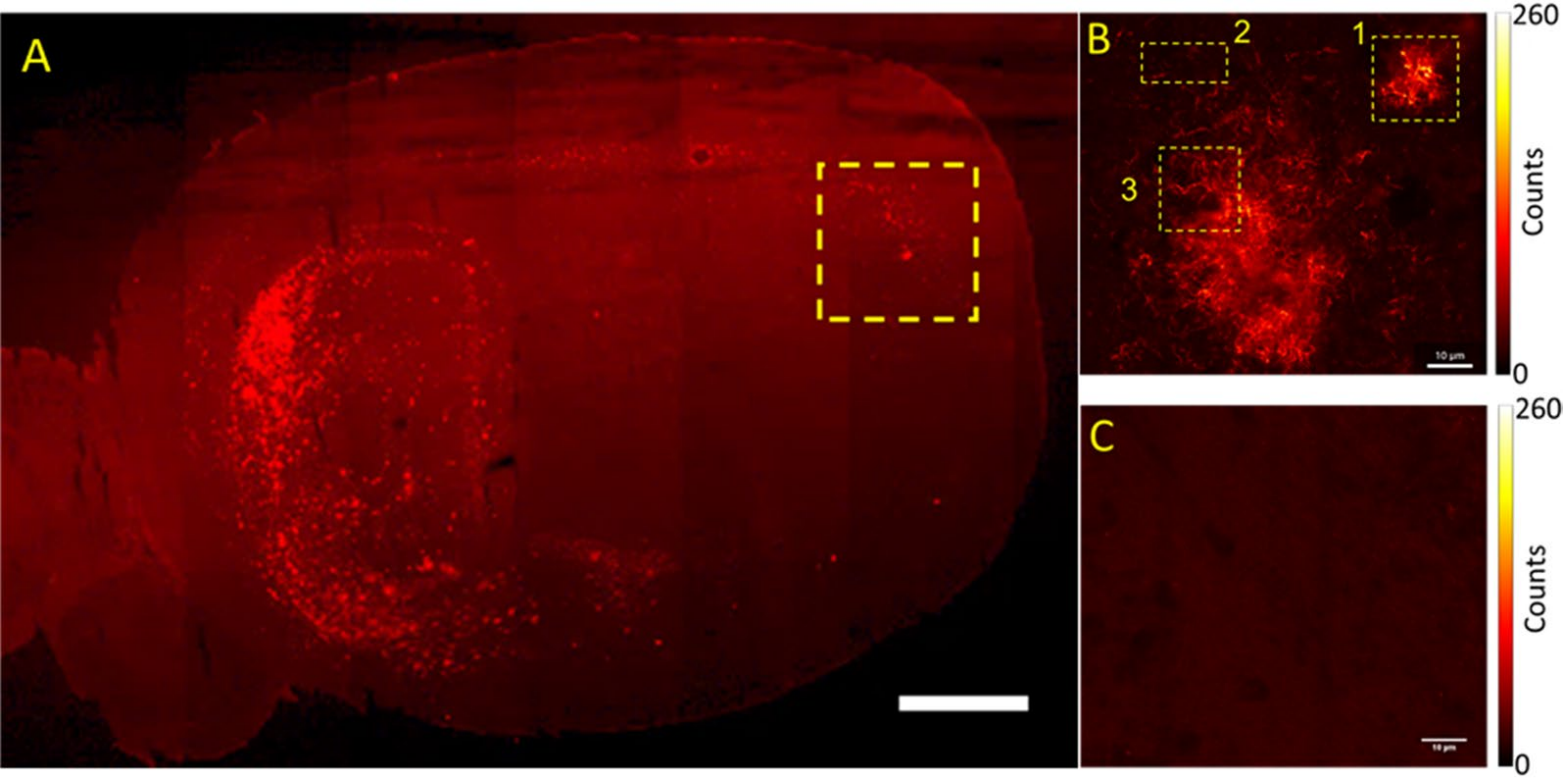
CLSM images of Aβ amyloidogenic aggregates in a brain tissue section from a 17 months old 3×TgAD mouse. **A** Large-capture tile & stich CLSM image of a sagittal mouse brain section showing Aβ amyloid aggregates in the hippocampus (left) and the cerebral cortex (right), visualized using 1C3-DyLight633. Scale bar 2 mm. **B** Magnified CLSM image of the rectangular area highlighted in A. Different regions selected for super-resolution STED microscopy imaging (shown in Fig. 3) indicate: a small plaque (1), plaque outskirt (2) and a detail of a large plaque (3). Scale bar 10 μm. **C** Immunohistochemical control obtained by displacing 1C3-DyLight633 by simultaneous incubation with unlabelled Aβ_40_ in large excess, (roughly 45 or 50 × higher). The average photon count in the background shown in C, PC_bg_ ≈ 7 photons, is the same as the background in B, indicating that: (1) nonspecific binding of 1C3-DyLight633 is low and (2) regions characterized by fluorescence > PC_bg_ indicate the presence of Aβ monomers/small-sized oligomers that could not be resolved by STED imaging. Scale bar 10 μm

Whole mouse brain tissue sections (Fig. 2A) showed distinct regions of Aβ aggregates’ accumulation (Fig. 2B) that were not observed in the negative control experiments, where antibody binding was blocked using a large excess (45–50 higher amount) of unlabelled Aβ_40_ peptide (Fig. 2C). The photon count level in the negative control (Fig. 2C) was similar in intensity to the level measured in the brain regions devoid of Aβ aggregate deposits, PC ≈ 7 photons. These results indicate that the 1C3 antibody against Aβ specifically recognizes Aβ aggregates in the mouse brain tissue, without giving rise to an increased background due to unspecific binding. This further implies that the fluorescence signal above the background level, which was observed around the clearly discerned fibrils in the Aβ plaque, is due to monomeric Aβ and possibly also small-sized Aβ oligomers that could not be discerned by STED microscopy.

**Fig. 3:**
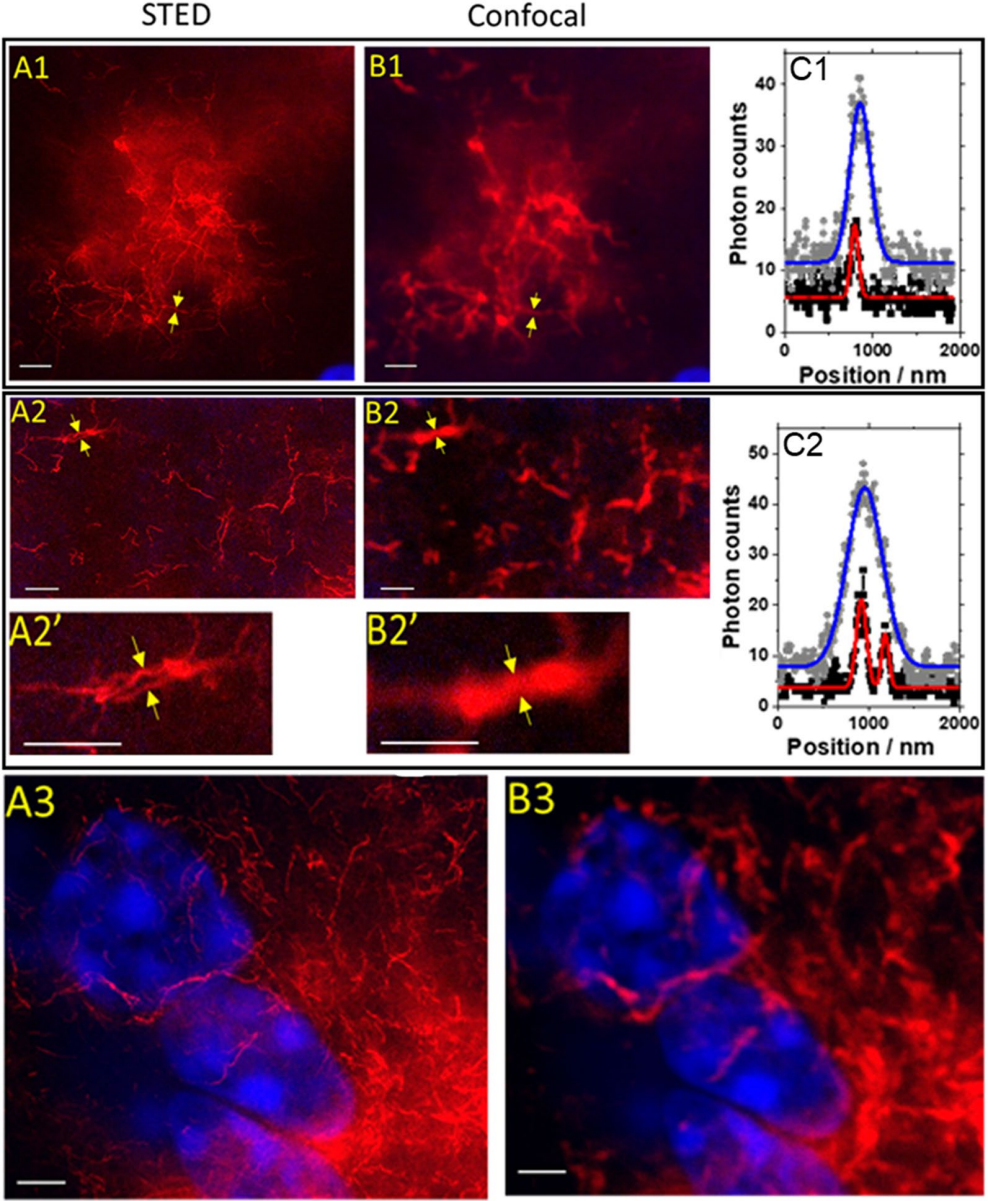
STED and CLSM images of Aβ amyloid aggregates in a brain tissue section from a 17 months old 3×TgAD mouse. **A1/B1** STED (**A1**) and CLSM (**B1**) images of a small plaque. The yellow arrows point to a detail at the plaque outskirt. **C1** Fluorescence intensity distribution profile shows that the detail from **A1**/**B1** is a single fiber with an apparent diameter of 87 nm measured by STED (black squares and corresponding Gaussian fit (red line)) and 320 nm by Confocal (grey diamonds and corresponding Gaussian fit (blue line)). **A2/B2** STED (**A2**) and CLSM (**B2**) images of a detail in the small plaque (magnified in A2’/B2’) showing bundled fibers that are clearly distinguishable by STED, but not by Confocal imaging. **C2** Fluorescence intensity distribution profile shows that STED could distinguish two fibers, 80 and 110 nm in diameter, that are 260 nm apart (black squares and corresponding Gaussian fit (red line)), whereas confocal imaging showed a blurred rod-like structure with a broad, single-peak fluorescence intensity distribution profile (grey diamonds and corresponding Gaussian fit (blue line)). **A3/B3** STED (**A3**) and CLSM (**B3**) images of a cell-dense area (DAPI nuclear stain (blue)), showing short fibrils in the perinuclear region and long fibrils located between cells that are hardly visible due to the large extracellular depositions of structured aggregates present nearby, where the 1C3-DyLight633 antibody binds in a large excess (red). Scale bar in all images is 2 μm

For further imaging with STED microscopy, we selected three brain regions where plaques were observed (Fig. 2B, yellow rectangles). The super-resolution STED images (Fig. 3A_1-3_, with corresponding CLSM images shown in Fig. 3B_1-3_), reveal that the smallest and the average fibril thickness determined by STED were 62 nm and (86 ± 18) nm, respectively (Fig. 3C_1_), compared to (320 ± 80) nm by CLSM. The STED images also showed that it was possible to successfully resolve two Aβ fibrils within a close distance, *d* = 260 nm, from one another (Fig. 3C_2_). Finally, dual-colour imaging with DAPI and 1C3-DyLight633 (Fig. 3A_3_ and B_3_), compellingly showed that STED microscopy can resolve individual Aβ amyloid fibrils in cell-dense regions neighbouring the plaque, as well as within the plaque.

## Discussion

This paper introduces super-resolution STED imaging of immunostained amyloid deposits in vitro (Fig. 1) and in brain tissue sections from an animal AD model (Fig. 3). STED microscopy enabled us to resolve details of the plaque structure that could not be resolved by confocal microscopy. Most notably, we were able to discern individual fibrils laying on top of one another in an untidy pile (Fig. 1A) that appeared by confocal imaging as a blurred diffuse smear (Fig. 1C) and measure their size—the average diameter of the in vitro formed fibrils was about (44 ± 13) nm in vitro, with the smallest diameter measured being 29 nm and the largest one 68 nm. The amyloid structures in brain tissue sections resolved in the present study are elongated, unbranched fibrils of an average fibril thickness of (86 ± 18) nm, ranging from 62 to 120 nm (Fig. 3), matching those in a recent in vitro STED study of an α-synuclein mutant with a 63-residue truncation in the N-terminal region that reported fine structures not resolved in confocal microscopy at a spatial resolution of 60–70 nm [25].

Based on the obtained results, several important STED microscopy applications are envisaged that could improve AD diagnostics and our understanding of basic mechanisms underlying AD.

Most notably, by pushing the boundaries of spatial resolution, Aβ tissue pathology can be examined at the nanoscale without resorting to EM. While indispensable in biomedical research—EM has been used to visualize with supreme spatial resolution details of synthetic amyloids structure in vitro, revealing the spacing between β-strands in a pleated β-sheet at a distance of 0.47 nm, protofilaments diameter of 3 nm, and fibrils of 8–10 nm in diameter (reviewed in [8]); has shown that different peptides/proteins, irrespective of their primary structure, chain length and native conformation, can acquire a prototypical, fibrillar amyloid structure [34–36]; and that amyloid filaments (dispersed to be suitable for cryo-EM reconstruction) may show AD-specific differences in interprotofilament packing [37], diagnostic EM is not widely available. The possibility to visualize Aβ tissue pathology at the nanoscale spatial resolution using STED microscopy is of relevance as the local environment in which peptides/proteins assemble into amyloid fibrils may significantly affect their morphology, giving rise to variations in twist periodicity, number of bundled filaments, sheet-like rather than ribbon-like assemblies etc. [38]. Hence, morphological analysis of plaques nanoscale structure may better reflect underlying AD pathology.

Optimal use of antibodies and other staining in AD diagnostics is far from settled. Comparison of traditionally used methods for Aβ amyloid plaque labelling, e.g. Congo red, Gallyas silver staining and Thioflavin-S, with one of the more commonly used antibodies for immunohistochemistry [39], antibody cross-reactivity analysis [40], and inter-laboratory comparison of neuropathological assessments of Aβ [41], showed good agreement in dichotomized valuations, presence/absence of Aβ plaques, but limited agreement in any more elaborate quantification analysis. In this context, the use of super-resolution microscopy and monoclonal antibodies that selectively reacts with Aβ aggregates, including soluble oligomers and insoluble fibrils, but do not bind to the monomers present in large excess, could be of high value as it could result in histopathological diagnostics that better reflect underlying pathology. Structured Aβ amyloid fibrils rather than unstructured protein aggregates are stable to the action of denaturing agents and proteases, and they have the mechanical strength of industrial materials [42]. The ability to observe individual Aβ amyloid fibrils with high resolution and sensitivity in relation to cell structures (Fig. 3A_3_), may enable studies on how amyloid fibrils cause damage to neighbouring cells. Studies of the anatomical relationship between fibrils and cell structure are important as there is evidence that amyloid fibrils can mechanically distort adjacent cells [43].

Spread of Aβ *via* neuro-anatomical pathways may be characterized in detail. For example, Armstrong and co-workers have compared the spatial patterns of amyloid deposits in sporadic AD and Down’s syndrome, finding that different disorders show considerable similarities in the spatial patterns of Aβ deposits, which may, in turn, suggest that the spread of Aβ *via* neuro-anatomical pathways may be common to several disorders [11]. However, differences were also observed among disorders. For example, the diffuse Aβ deposits were more frequently distributed in regular clusters in AD, while cluster sizes of the diffuse and primitive deposits were significantly smaller in chronic traumatic encephalopathy [11]. Results from intracerebral injection of Aβ-rich brain extracts suggest that Aβ aggregation can be initiated by seeding [44]. Interestingly, it has been found that tau misfolding can propagate between individual hippocampal neurons [45]. The STED methodology can be useful for studies of such propagation, and it will be interesting to know whether similar transfer holds for Aβ, as network patterns of Aβ deposition have been described in Parkinson’s disease [46]. Astrocytes may also play a role, as there is evidence for toxic Aβ oligomers induced self-replication in astrocytes triggering neuronal injury [47, 48] developed spatially extended nucleation-aggregation-fragmentation models for the dynamics of prion-like neurodegenerative protein-spreading in the brain. The prion-like hypothesis of neurodegenerative diseases states that the accumulation of misfolded proteins in the form of aggregates is responsible for tissue death and associated neurodegenerative pathology and cognitive decline. The spreading and aggregation of both Aβ and tau molecules in the brain connectome has recently been modelled [49]. In a mouse model, a “feed-forward” mechanism whereby Aβ plaques enhance endogenous α-synuclein seeding and spreading over time post-injection has been proposed [50].

Finally, recent success in the development of immunotherapies against Aβ for the treatment of AD that were shown to reduce the amyloid load [28], underline the need for observing at the nanoscale spatial resolution local plaque clearance mechanisms while monitoring the therapeutic efficacy of these treatments. STED microscopy may also shed light on our understanding of mechanisms through which negative side effects may arise [44] and may also help us understand how amyloid deposition builds-up/is cleared within cerebral vessels in cerebral amyloid angiopathy (CAA).

## Data Availability

All data and related analyses are included in this published article. All other data is available from the corresponding author upon request.
